# Effect of Pr_2_O_3_ addition on the mechanical properties of the mullite/ZTA composites

**DOI:** 10.1038/s41598-026-43191-7

**Published:** 2026-04-02

**Authors:** S. M. Naga, M. Awaad, A. A. Amer, M. Arif, M. A. Saleh, M. Elshaer

**Affiliations:** 1 Ceramics Department, National Research Centre, El-Bohous Street, Cairo, 12622 Egypt; 2 Chemistry Department, Faculty of Science, Zagazig University, Zagazig, Egypt; 3 Technological Military College, Cairo, Egypt; 4 Technical Research Centre, Cairo, Egypt

**Keywords:** Pr_2_O_3_, Mullite, ZTA, Kaolinite, Mechanical properties, Engineering, Materials science

## Abstract

To enhance the mechanical and physical properties of Mullite/Zirconia Toughened Alumina (ZTA) composites for advanced engineering applications, rare-earth elements are introduced as dopants. Such composites are characterized by high hardness, fracture toughness, thermal shock resistance, and enhanced physical properties thanks to controlled microstructure through stabilization of the ZrO_2_ phases, enabling the formation of rod-like particles to enhance mechanical characteristics. In the present study, mullite/ ZTA) doped with 0.5, 0.75, and 1 wt% Pr_2_O_3_ and a complete study of their effects on the produced composites were carried out. All batches were dry mixed and shaped using a cold isostatic pressing (CIP) at 300 MPa. The specimens were then pressureless sintered at 1550–1650 °C for two hours, with 50 °C intervals and 5 °C/min firing and cooling rates. Microstructure and phase composition of the sintered ceramic composites were evaluated. Vickers hardness, flexural strength, and fracture toughness tests were used to determine the mechanical properties of the ceramic composites. The densification behavior, microstructure, and mechanical properties of the (mullite/ZTA) composites were found to be significantly impacted by an increase in Pr_2_O_3_ concentration.

## Introduction

Special, technical, or engineering ceramics are referred to by advanced ceramics. It is important to mention that “advanced materials” refers to a combination of material, process, product, and application rather than just the material itself. They have better mechanical properties, resistance to oxidation and corrosion, as well as electrical, optical, and/or magnetic properties. Aluminum oxide, mullite, zirconium oxide, neodymium oxide and praseodymium oxide are among the various advanced ceramic materials. Among advanced ceramics, aluminum oxide (Al_2_O_3_) ceramics are currently the most widely used and cost-effective structural engineering material^1^. It is the perfect material to serve effectively in a range of harsh settings, including mining and chemical industries, metal manufacture and processing, ceramic armors, and biomedical applications, due to its high hardness, abrasion resistance, and chemical inertness. The primary drawback of aluminum oxide sintered bodies is their low fracture toughness.

The excellent mechanical and thermal properties of zirconia toughened alumina (ZTA) ceramics—such as high strength, dimensional stability, high-temperature strength, hardness, wear resistance, and thermal resistivity—make them ideal for a wide range of applications^2–4^. The improvement of the characteristics of ZTA composites has been the focus of numerous researches. Therefore, methods to increase the fracture toughness of ZTA composites have been investigated, including oxide addition, sintering modulation, and the creation of alternate synthetic pathways^5^. The second additive addition can specifically lower the sintering temperature, modify the microstructure, and improve the mechanical and thermal properties of ZTA ceramics.

On mixing ZTA ceramics with a small addition of sintering aids, such as MgO, kaolin, and La_2_O_3_, ZTA densification could be achieved at low temperatures^6^.

On the other hand, some rare earth elements were added to ZTA composites to enhance their mechanical properties. Niobium pentoxide (Nb_2_O_5_) and titanium oxide (TiO_2_) were added to zirconia toughened alumina mixed with MgO to improve the fracture toughness of the ceramic composite. While the sintering temperature is reduced, the mechanical and physical properties of the produced composites are improved. Additions of 3 wt% Nb_2_O_5_ and TiO_2_ were found to be optimal; the bulk density, fracture toughness, and hardness showed the highest values, while porosity exhibited the minimum value. At 3% Nb_2_O_5_ and TiO_2_, the ideal values for density, hardness, and fracture toughness were determined to be 4.102 g/cm^3^, 1145.6 H_V_, and 9.36 MPa.m^1/2^, respectively^7^.

A study carried out by Sktani et al.^8^, revealed the impact of La_2_O_3_ inclusion (0–3 wt% %) on the mechanical properties, phase formation, and microstructure of ZTA ceramics reinforced with 5.0 wt% CeO_2_. Their results proved that the formation of LaAl_11_O_18_ triggered the phase transformation, densification of the ZTA-CeO_2_ matrix, microstructure refinement, and sintering. The study results demonstrated that the porosity reduction, ZTA-CeO_2_ composite densification, and Al_2_O_3_ grain refining a rapid increase in hardness of ZTA-CeO_2_ composites are noticed. The authors claimed that the in-situ production of elongated LaAl_11_O_18_ grains improves fracture toughness. The ideal results for density (4.41 g/cm^3^), porosity (0.46%), hardness (1792 HV), and fracture toughness (8.8 MPa.m^1/2^) were obtained with the addition of 0.7% La_2_O_3_. However, they found that too much La_2_O_3_ is harmful since it exhibits poor mechanical properties because of the coarsening of Al_2_O_3_ grains, the decrease in density, and the poor compactness of many LaAl_11_O_18_ grains.

It was reported that the formation of elongated LaMgAl_11_O_19_ promoted the mechanical properties of the ZTA composites^9^. On the other hand, the addition of La_2_O_3_ and Y_2_O_3_ reduced the sintering temperature of the ZTA composite and enhanced its mechanical properties^10^. In a study conducted by Xu et al.^11^ they reported that the addition of 1.5 wt% La_2_O_3_ constructed LaAl_11_O_18_ particles. The formed particles enhanced the fracture toughness through crack bridging and grain pullout mechanisms. In addition, the presence of the LaAl_11_O_18_ boosted the densification parameters. They showed that the preparation of ZTA composites containing La_2_O_3_ as an additive enhanced both the flexural strength and the thermal shock resistance of the produced composites.

Praseodymium aluminide (PrAl_2_) is used also, to enhance the fracture toughness of the ZTA composites. The key to the toughening effect of PrAl_2_ plate-like crystal on ZTA is the temperature differential between the growing temperature of the plate-like crystals and the sintering temperature of the ZTA matrix. The PrAlO_2_ precursor used in this study was fabricated by combustion, and highly active PrAlO_2_ was created via pre-sintering at 900 °C. The process above yielded plate-like crystals with a high aspect ratio, which contributed to toughening by creating a 100 °C difference between the growth temperature of the plate-like crystals and the ZTA sintering temperature. In addition, the solid dissolution of Praseodymium ions into ZrO_2_ slows down the hydrothermal ageing process of ZrO_2_, reduces phase transformation-induced microcracks, and increases ZTA’s fracture toughness and Vickers hardness. The findings demonstrate that, in comparison to ZTA, the Vickers hardness improved from 18.95 GPa to 19.39 GPa, the fracture strength increased from 654.78 MPa to 687.59 MPa, and the fracture toughness of ZTA reinforced and toughened by Pr_0.833_Al_11.833_O_19_ plate crystal in a human environment simulation (hydrothermal aging) increased from 7.13 MPa·m^1/2^ to 8.97 MPa·m^1/2^^12^.

A study was conducted to assess how the formation of in-situ mullite whiskers affects the properties of mullite/3Y-TZP composites. The mullite was synthesized in situ by incorporating varying amounts of tourmaline into 3Y-TZP. It was noted that the size of the added tourmaline particles affected the diameter of the formed mullite whiskers. The diameter of the whiskers varied from 90 nm to 540 nm as the tourmaline particle size changed from 125 nm to 900 nm. The best mechanical properties were achieved with tourmaline particles measuring 330 nm in size^13^.

In fact, we examined the related literature during the last 20 years. Nearly all research articles have investigated the effect of adding rare-earth elements to alumina, zirconia and ZTA. The impact of praseodymium oxide (Pr_2_O_3_) doping on the behavior of ceramic composites is still not well established. The current study specifically examines the effect of Pr_2_O_3_ doping on the physical, phase composition, microstructure, and mechanical properties of mullite/zirconia/alumina composites doped with Pr_2_O_3_. To control the densification behavior and the microstructure and mechanical characteristics of the composite, the sintering temperature was first determined. Then the composites were doped with 0.5, 0.75, and 1 wt% of Pr_2_O_3_, and the doped composites’ full characteristics were then evaluated.

## Materials and methods

### Materials

The used starting materials were Alumina (α-Al_2_O_3_) (D50 = 10 μm, provided by Silkem Co., Slovenia), plastic kaolin (D50 = 2 μm provided by El Nasr Mining Co., Egypt), TZ-3Y-E partially stabilized zirconia powder with a grain size of 50 nm and a uniform dispersion of 3 mol% yttria (provided by Tosoh Co., Tokyo, Japan), Praseodymium oxide (99.9% Pr_2_O_3_, Strem chemicals, Newburyport, USA), and chemically pure magnesium oxide with purity of 99.5% (NEI CO., USA).

### Methods

#### (a) Batch formulation

The batch composition of the composites under study is shown in Table 1. Every batch was calculated on a weight% basis. Every batch was ball milled for five hours using ball mill at 200 rpm. The batches were shaped into bars with dimensions of 5 × 5 × 60 mm for mechanical evaluation and into pellets with 10 mm in diameter and 3 mm in thickness to follow up densification characteristics. The samples were manufactured using cold isostatic pressing (CIP) at 300 MPa. For two hours, the pellets were pressureless sintered at 1550, 1600, and 1650 °C with a 5 °C/min heating and cooling rate.

**Table 1:** Batch composition for the studied composites.

Batch no.	AL_2_O_3_, wt%	Kaolin, wt%	ZrO_2_, wt%	MgO, wt%	Pr_2_O_3_, wt%
M0	75	10	15	0.5	0
P1	75	10	15	0.5	0.5
P2	75	10	15	0.5	0.75
P3	75	10	15	0.5	1

#### (b) Characterization

The water displacement method was used to determine the physical parameters in terms of bulk density and apparent porosity of the sintered ceramic specimens, following *ASTM C20*. Vickers hardness was assessed by a hardness tester (Omnimet automatic MHK system Model MicroMet5114, Buehler USA) on mirror-polished surfaces following *ASTM C1327-99*. Indentations were made on the polished surfaces with a load of 5 kg and 15-s dwell time. 30 indents were made for each sample. The flexural strength of the samples was determined by the methods described in *ASTM C1161-02* using a universal testing machine (Testometric, model M350-10 A, UK). The fracture toughness was measured via a single-edge V-notched beam (SEVNB) technique following *ASTM C1421-01* using 3- point bending fixture. The phase composition and microstructure of the sintered bodies were examined with a Philips x-ray diffractometer (model PW1730, Eindhoven, the Netherlands) equipped with a Cu target and a Ni filter and a scanning electron microscope (model XL 30, Philips, Eindhoven, Netherlands) equipped with an energy dispersive x-ray spectrometer (EDS).

## Results and discussion

### Physical properties

**Fig. 1:**
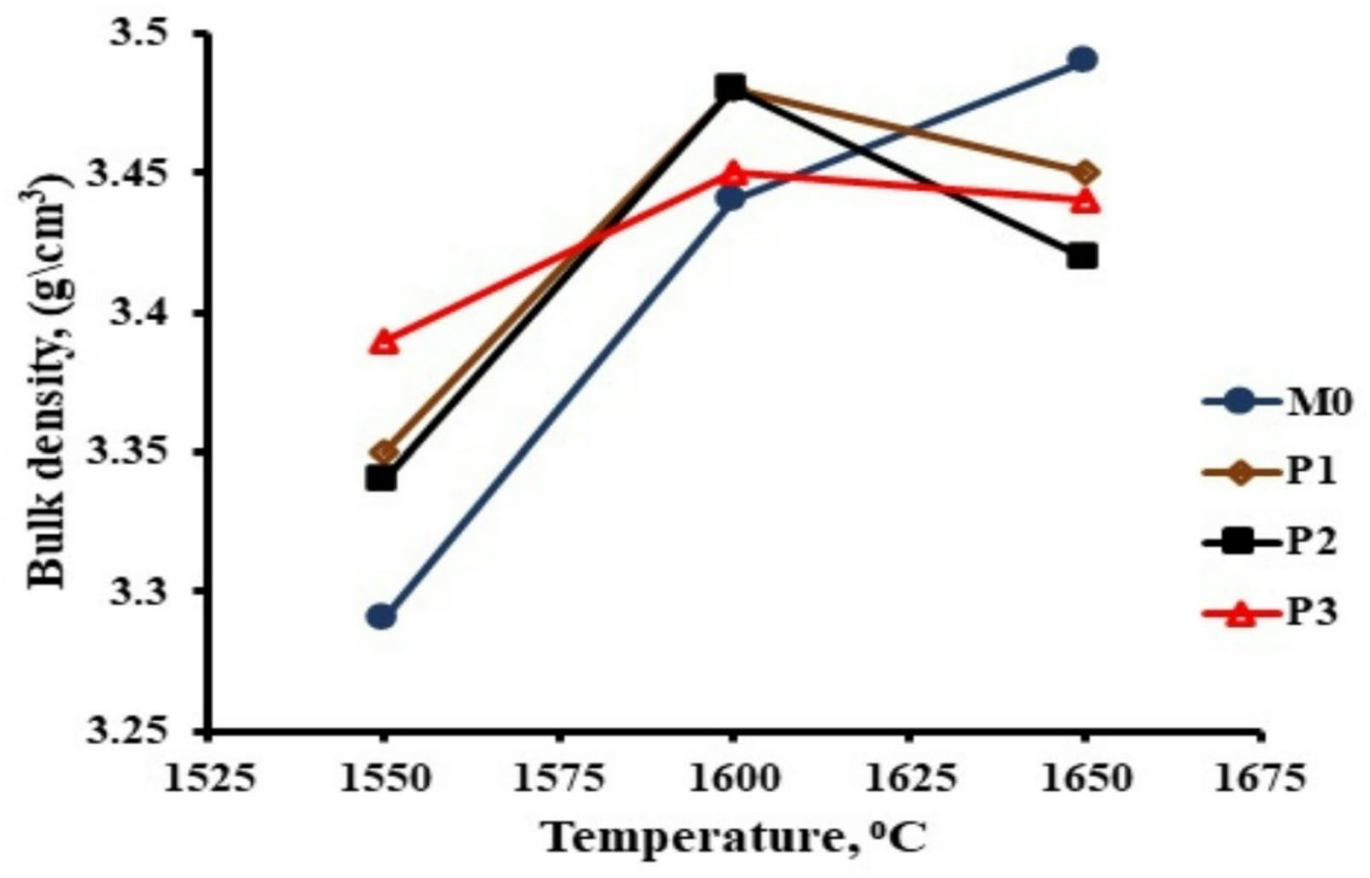
Bulk density of studied composites sintered at different sintering temperatures.

The effect of Pr_2_O_3_ addition on the sinterability of the studied composites is illustrated in Fig. 1. Pr_2_O_3_- free composites showed a higher sintering temperature of 1650 °C as compared to those containing different amounts of Pr_2_O_3_. The effect of addition of Pr_2_O_3_ (from 0.5 to 1wt. %) has clearly demonstrated in decreasing both the sintering temperature and the bulk density. The sintering temperature of the doped samples was lowered by 50 °C compared to the undoped sample. As expected, the increase in the bulk density was accompanied by a decrease in the apparent porosity up to the optimum sintering temperature. The increase in the porosity and decrease in density of samples containing 0.75% Pr_2_O_3_ with increasing sintering temperature up to 1650 °C may be due to the formation of closed pores (a phenomenon that had already been observed in the overfired ceramic bodies).

The ionic radius of Pr^3+^ is 185 nm, and that for Zr^4+^ is 0.072 nm, which is similar to the situation on doping Zr with Ca ions^2^. On doping ZTA composites with Pr^3+^, which has a larger ionic radius, the lattice parameters will increase^14^. In fact, such enlargement is partially counteracted by oxygen vacancy substitution for charge neutrality conservation^15^. Doping with a small Pr^3+^ percentage leads to a small oxygen vacancy, while higher doping percentages cause agglomeration and larger vacancies^16^. The substitution of Zr^4+^ in the lattice by Pr^3+^ is illustrated by the following Kroger-Vink notation:

$${\mathrm{Z}}{{\mathrm{r}}^{\mathrm{X}}}_{{{\mathrm{zr}}}}+{\text{ }}{{\mathrm{O}}^{\mathrm{X}}}_{0}\overset {} \longleftrightarrow {\mathrm{Ca}}{\hbox{''}_{{\mathrm{zr}}}}+{\text{ }}{{\mathrm{V}}_{{\"O }}}$$

Ca^’’^_zr_ represents that C^2+^ occupies the site normally occupied by a Zr^4+^ion; V_Ö_ represents an oxygen vacancy.

**Fig. 2:**
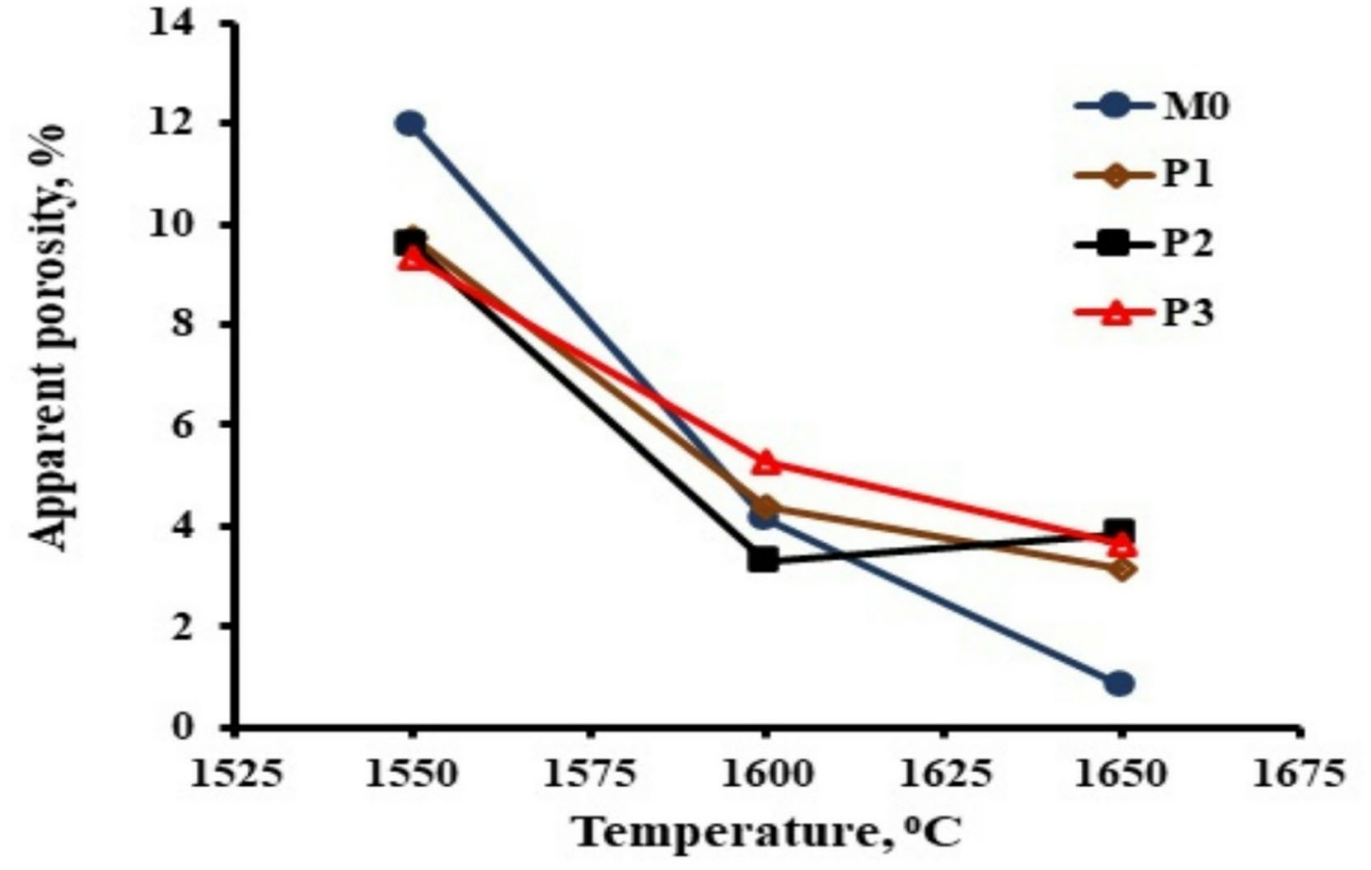
Apparent porosity of studied composites sintered at different sintering temperatures.

It is articulated that lower charge state, larger ionic size, and ionicity favor the stabilization of Zr^4+^^17^. As Pr^3+^ exhibited all the above-mentioned features, we believe that it will help in stabilizing the high-temperature phases of zirconia (Fig. 2).

###  Phase composition

The phase composition study of the samples (using XRD analysis) indicates that the samples are composed of alumina, mullite, tetragonal and monoclinic zirconia. It is believed that the addition of kaolin, with its silica content, contributes to the formation of the mullite phase. As expected and due to the presence of low amount of silica, the formed mullite content was limited. The main phases were alumina and zirconia. Pr_2_O_3_ was not identified in the XRD pattern, even in the highest amount of 1 wt%. It might have been consumed in the formation of the glassy phase, or its content was under the detection limit of the XRD instrument. The percentages of monoclinic and tetragonal zirconia, were illustrated in Table 2. It showed that the increase in Pr_2_O_3_ content from 0.5 up to 0.75 wt% enhanced the t-→m- ZrO_2_ transformation. More Pr_2_O_3_ addition (1 wt%) reduced the ability of t→m ZrO_2_ transformation.

**Fig. 3:**
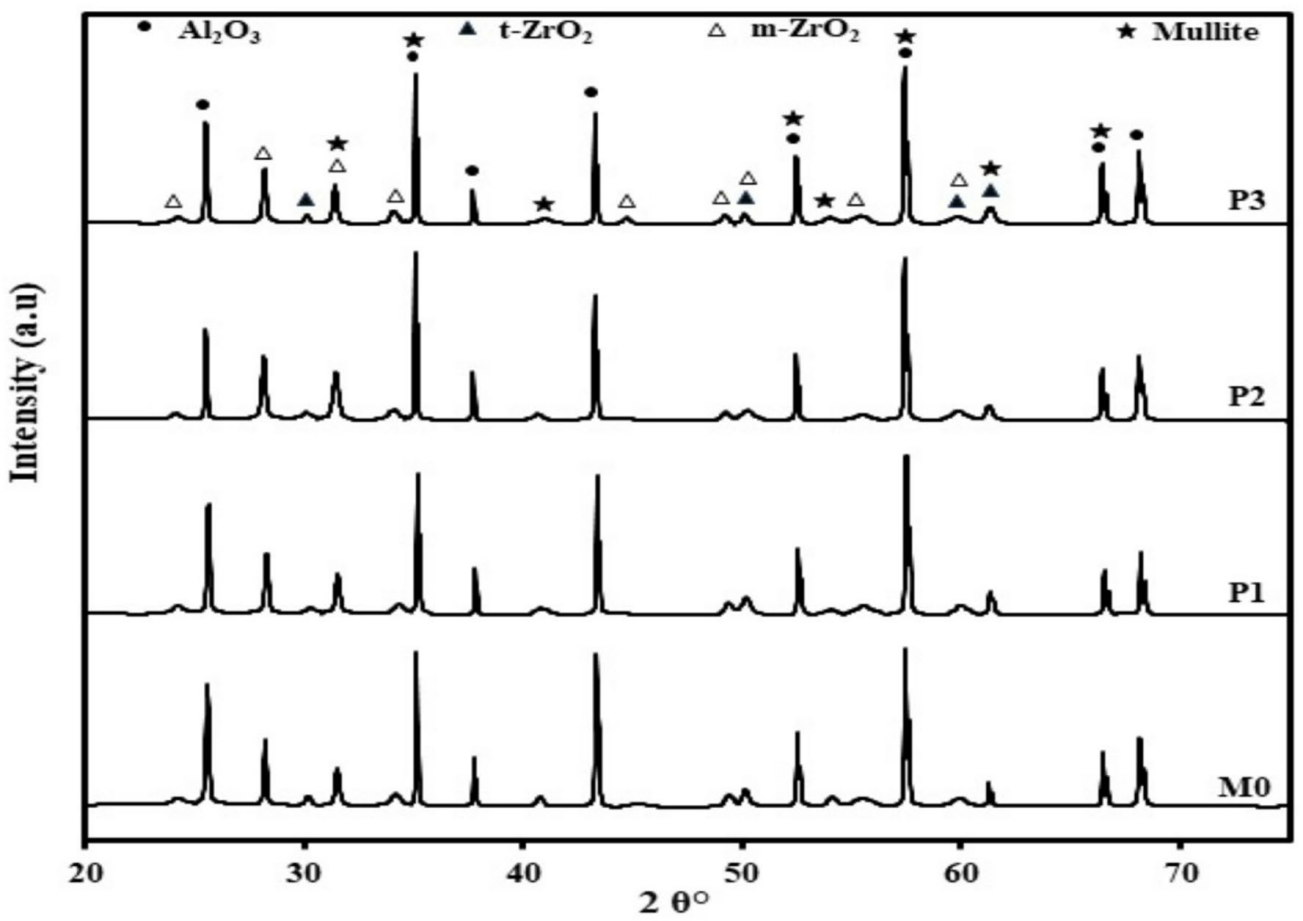
XRD patterns of the of studied composites sintered at 1600 °C.

It was stated that the most affected aspects controlling the t→m ZrO_2_ transformation are lattice dislocations^18^ and intrinsic defects^19^. It was demonstrated that O_2_ vacancies reduce the Zr valence state from Zr^+^4 to Zr^+ 3^ or Zr^+ 2^^20^. Such a reduction supports the stabilization of t-ZrO_2_. On the other hand, oxygen vacancies facilitate the stabilization of t-ZrO_2_ by lowering the energy required for stabilization. The presence of trivalent, tetravalent, or pentavalent^21–23^ dopants create oxygen vacancies and, as a result, enhances the stabilization of t-ZrO_2_. We believe that the monoclinic content decreases by 1 wt% Pr_2_O_3_ addition due to the increase in O_2_ vacancies stimulated by the high percentage of trivalent Pr^3+^ ions. (Fig. 3)

**Table 2:** Tetragonality and Monoclonality % of different studies composites.

Batch no.	Tetragonality, %	Monoclonality, %
M0	11.27	88.73
P1	8.58	91.42
P2	7.47	92.53
P3	12.16	87.84

###  Microstructure

**Fig. 4:**
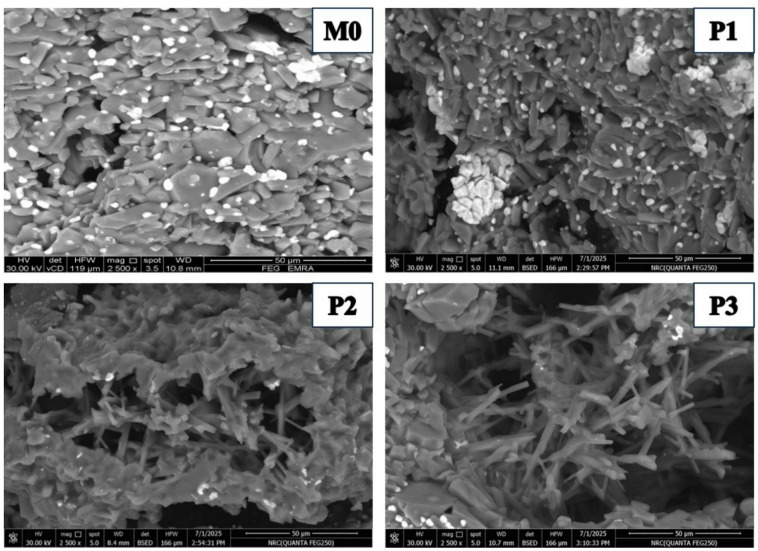
Demonstrated the general view of the 4 batches under study.

Figure 4 demonstrates the general microstructure of the studied batches M0, P1, P2, and P3 batches; denoted by a, b, c, and d, respectively. The figure reveals that increasing Pr_2_O_3_ content promotes the formation of rod-like alumina grains and ZrO_2_ grains typically retain a finer grain size without agglomeration. They are present both in and around the alumina grains. The ZTA blank composite, as shown in Fig. 4a, indicates that zirconia particles are distributed homogeneously all over the matrix. ZrO_2_ grains are present as either rounded small grains that are present at the triple point of the alumina particles, which inhibits the alumina grains’ growth, or they are partially agglomerated and mostly located in the grain’s triple junction. It is reported that enclosing the Al_2_O_3_ grain boundaries with ZrO_2_ fine particles hinders the Al_2_O_3_ grain boundary immigration and deprives the Al_2_O_3_ grain growth^24,25^.

Many authors reported that the surface energy of t-ZrO_2_ is higher than that of m-ZrO_2_, due to the effect of grain size on phase stability. It is documented by Garvie^26^ that, with a decrease in grain size, the overall free energy effect on the stability of the grains increases significantly. Figure 4(b, c and d) shows the impact of Pr_2_O_3_ addition on the t→ZrO_2_ transformation. We believe that increasing Pr_2_O_3_ content beyond 0.75 wt% had a harmful effect on the stability of the monoclinic ZrO_2_ phase. Anyway, a more detailed study is needed for such a case.

**Fig. 5:**
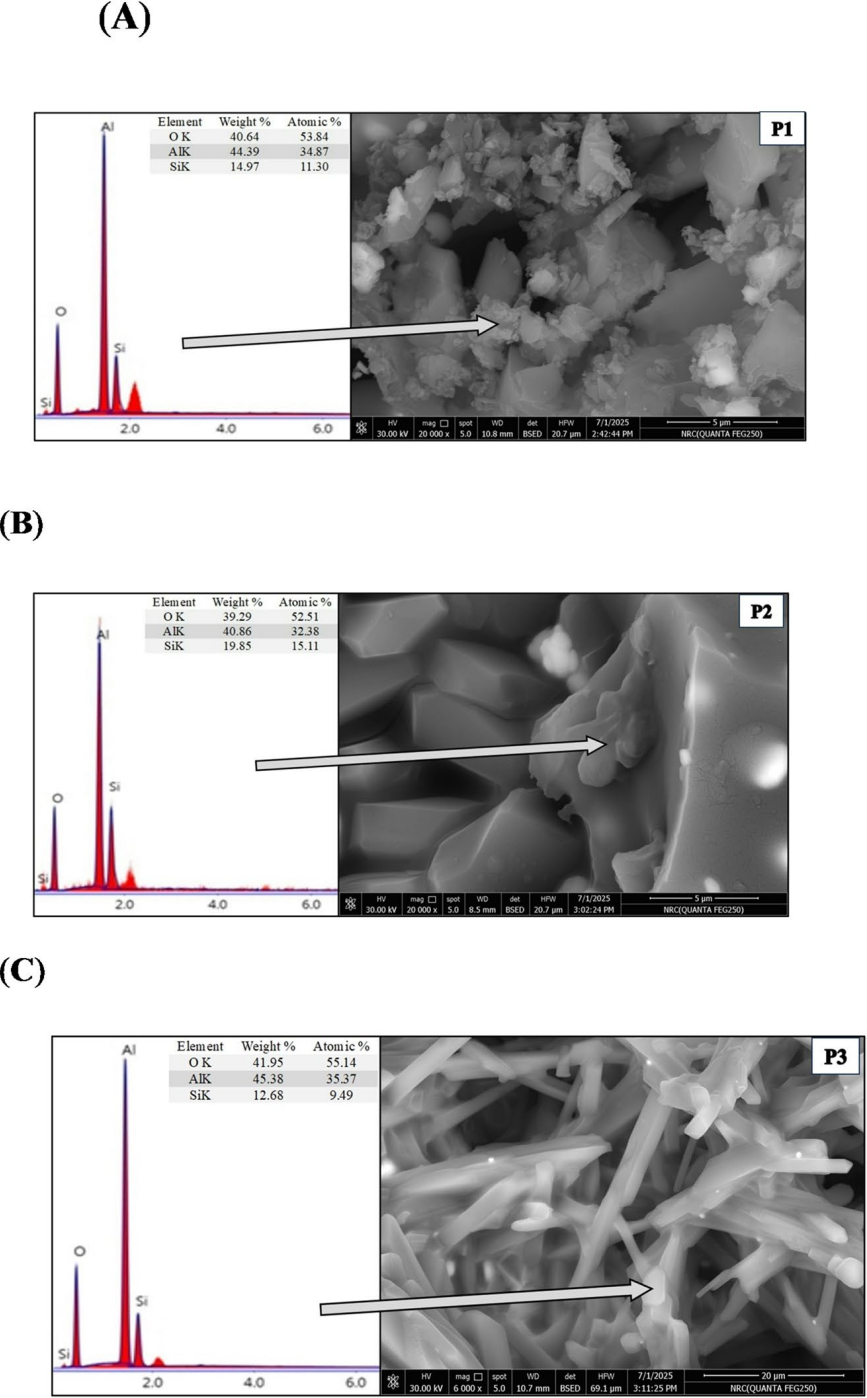
**(a)** Mullite formation in the sintered P1 sample. **(b)** Mullite formation in the sintered P2 sample. (c): Mullite formation in the sintered P3 sample.

The presence of mullite was indicated by the SEM findings as well as the XRD results. Figures 5a–c exhibited the samples microstructure and their relative EDS analyses. Samples P1 and P2 showed flake-like mullite grains. The higher the concentration of Pr_2_O_3,_ the coarser the mullite grain size. On the other hand, the increase in the Pr_2_O_3_ addition to 1 wt% (P3 sample) showed relatively coarse, rounded mullite grains, and both P2 and P3 samples showed the presence of thin rod-like mullite grains.

**Fig. 6:**
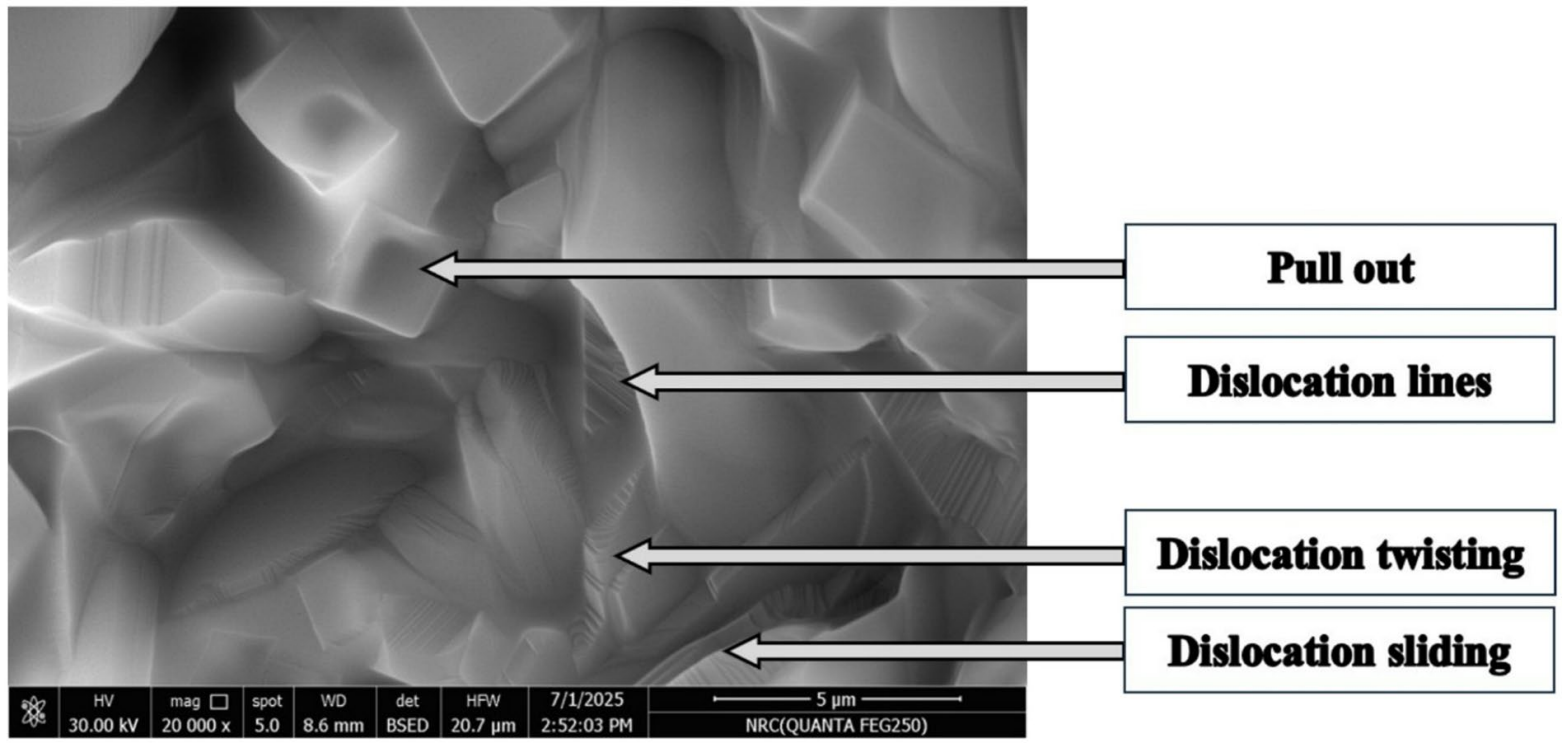
Line, rings, twisting, and sliding dislocation (P2).

It was noticed that alumina grains in both P1 and P3 are nearly similar in shape and size. They have different grain sizes; some of them are coalescence to generate alumina grain growth, which is associated with grain boundary migration, while others were characterized by their equiaxed shape of varying sizes. P2 sample exhibited an additional microstructure feature represented in dislocation line, dislocation rings, dislocation twisting, and dislocation sliding, which were marked within the alumina grains, Fig. 6^27–29^. Figure 5b showed the presence of plate-like alumina grains of the P2 sample, where ZrO_2_ are either embedded in or found in the triple junction of the grains.

###  Mechanical properties

#### (a) Fracture toughness (K_ic_)

The fracture toughness of the sintered samples tends to increase with the increase of the addition of Pr_2_O_3_ as illustrated in Fg. 7. The figure showed an increase in the K_ic_ with increasing Pr_2_O_3_ up to 0.75 wt%, then it started to slightly decrease with increasing the Pr_2_O_3_ wt% to 1%. It may be due to the increase in the apparent porosity of sample P3, Fig. 2. Another factor that can affect the fracture toughness of the sample P3 is its reduction in the monoclinic zirconia content, Table (2). It is well known that the m-ZrO_2_ increases the fracture toughness through transformation toughening mechanism. In such a mechanism, and when a crack was formed and propagated, m-ZrO_2_ is created under stress, leading to localized compressive forces, which could stop or slow down the crack growth. The other factors for enhancing the fracture toughness in the samples are the formation of Al_2_O_3_ anisotropic elongated grains (Fig. 4), which can generate other toughening mechanisms such as crack bridging Fig. 8 and crack deflection Fig. 9. The enhancement in the fracture toughness of P2 might also be attributed to dislocation lines, dislocation rings, dislocation twisting and sliding in the Al_2_O_3_ matrix (Fig. 6). It is assumed that in such a mechanism, the elastic strain energy is settled down in the dislocation, which puts the dislocation in a sub-stable state. It will proceed as follows: on reaching the crack, the dislocation will be able to absorb part of the fracture’s energy via its deformation. Accordingly, the crack propagation will be firmed and the fracture strength enhanced^30^. Figure 6 demonstrates the micrograph of alumina grains with pull-out of ZrO_2_ grains. Pull-out mechanism is a well-known toughening mechanism of the ceramic composites. In such a mechanism, the grains or fibers ingrained in the matrix or other grains are pulled out, absorbing energy and increasing the material’s toughness. Accordingly, it is assumed that the combination of the plate-like alumina grains, rod-like mullite grains, the dislocation lines, dislocation rings, dislocation twisting and sliding in the Al_2_O_3_ matrix, and the pull-out Zr_2_O_3_ grains are the essential aspects that augmented the fracture toughness of Pr_2_O_3_-doped composites.

**Fig. 7:**
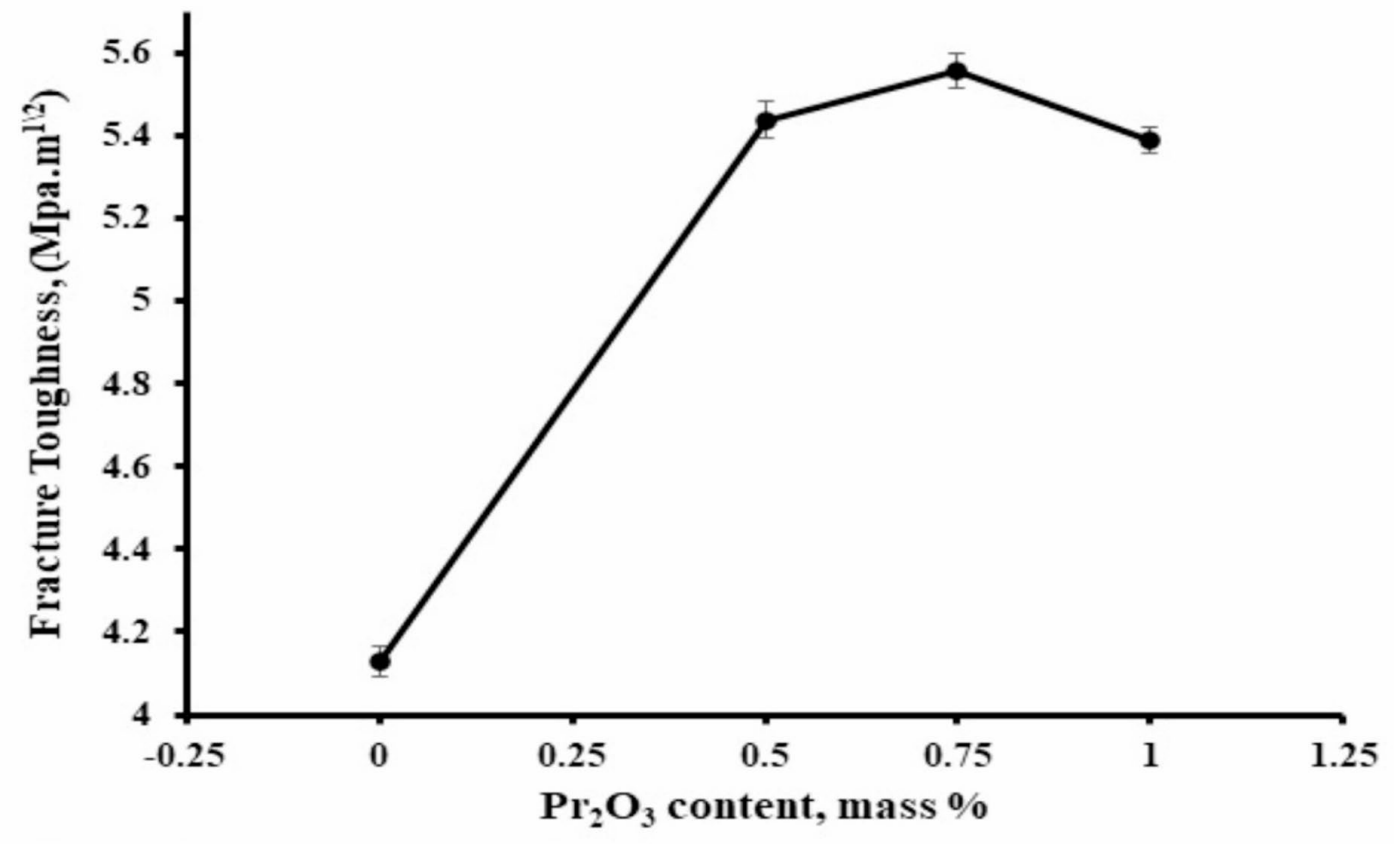
Fracture Toughness of sintered composites.

**Fig. 8:**
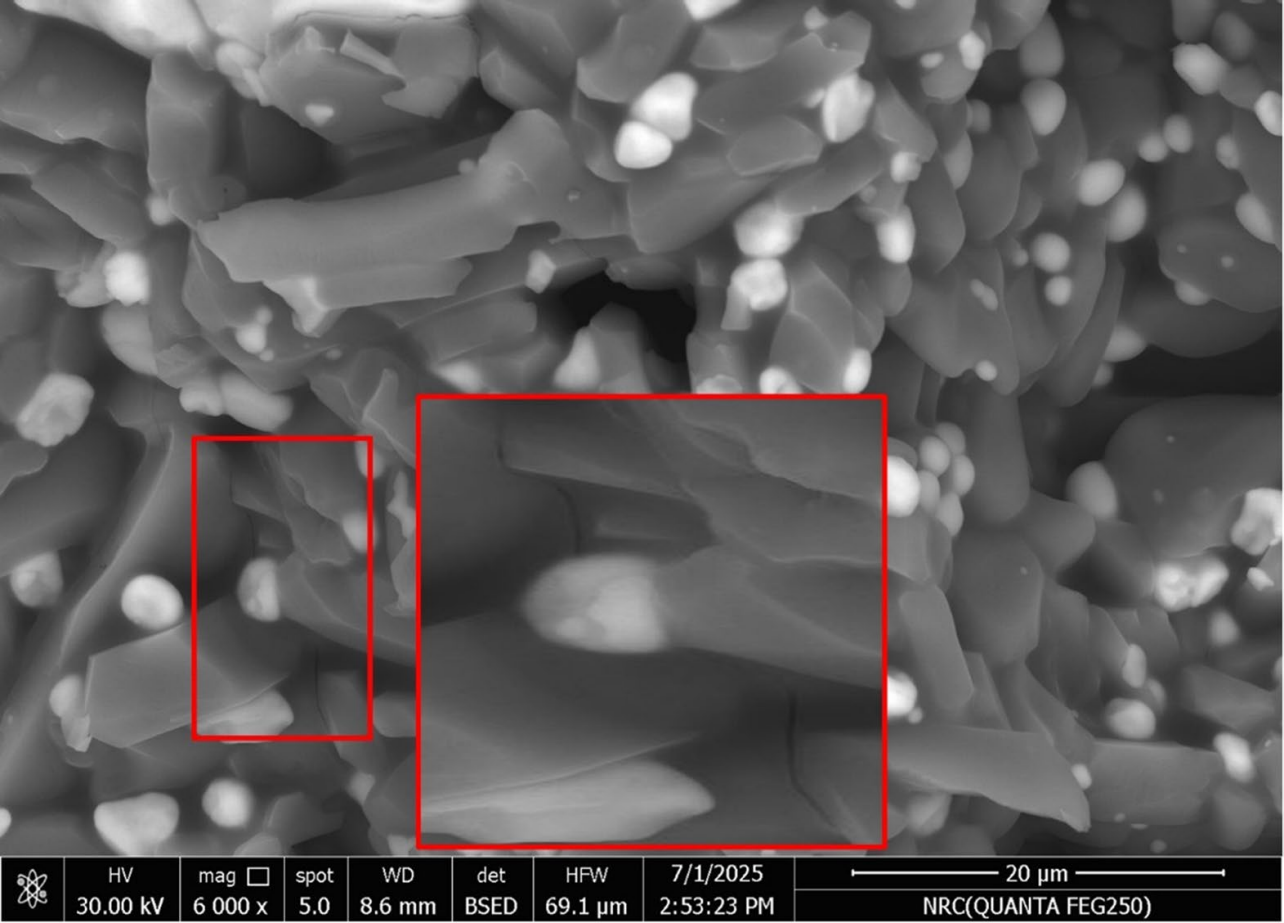
Crack bridging in the sintered P2 sample.

**Fig. 9:**
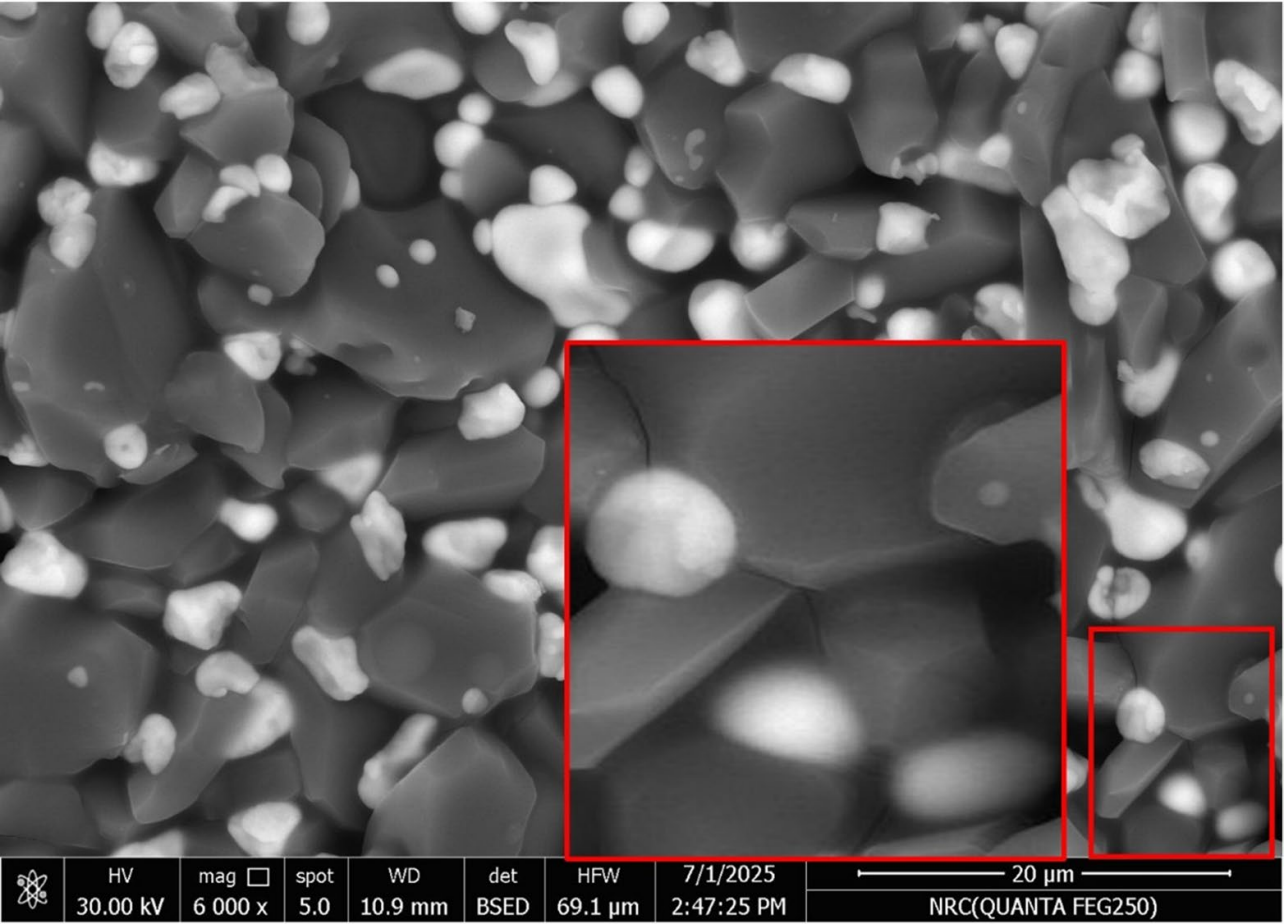
Crack deflection in the sintered P1 sample.

Praseodymium in the form of Pr_0.833_Al_11.833_O_19_ was also used to enhance the fracture toughness of the ZTA composites. The legend of the toughening effect of the praseodymium aluminate returns to the temperature differential between the growing temperature of the Pr_0.833_Al_11.833_O_19_ plate-like crystals and the sintering temperature of the ZTA matrix^12^. The M-type praseodymium aluminate used in this study was fabricated by combustion, and highly active praseodymium aluminate was created via pre-sintering at 900 °C. The process above yielded plate-like crystals with a high aspect ratio, which contributed to toughening by creating a 100 °C difference between the growth temperature of the plate-like crystals and the ZTA sintering temperature. In addition, the solid dissolution of Praseodymium ions into ZrO_2_ slows down the hydrothermal ageing process of ZrO_2_, reduces phase transformation-induced microcracks, and increases ZTA’s fracture toughness and Vickers hardness^12^.

#### (b) Flexural strength and Vickers hardness evaluation

**Fig. 10:**
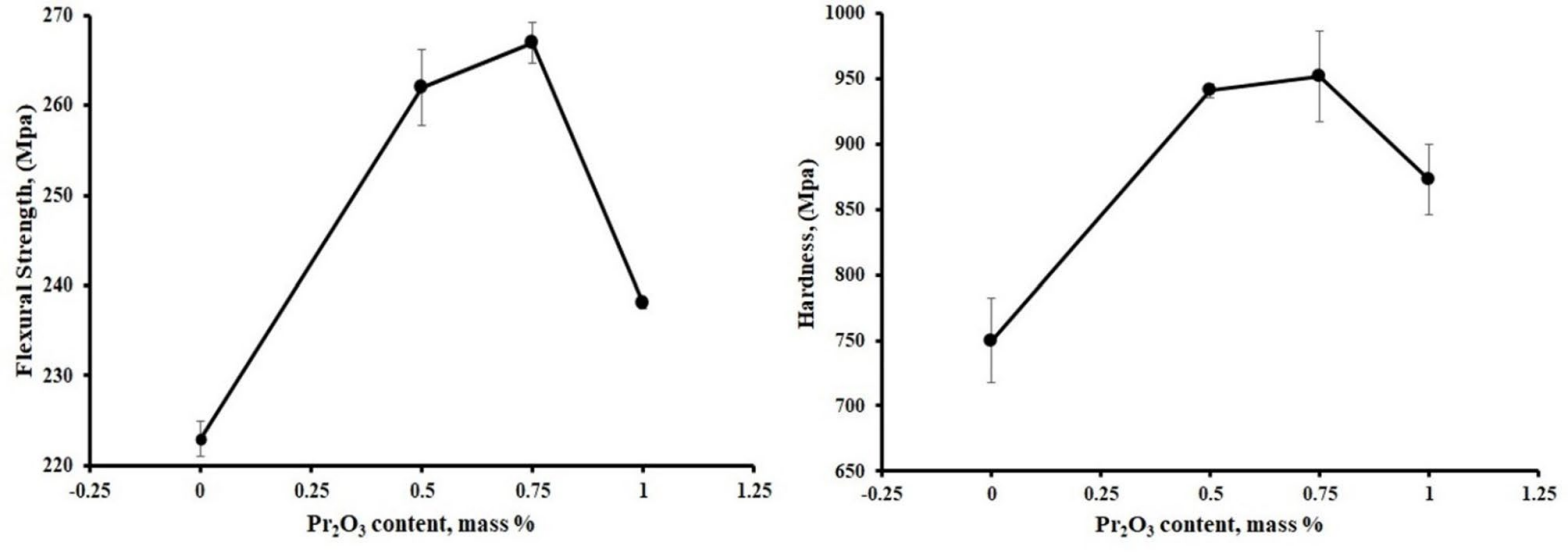
(**a**) Flexural strength, (**b**) hardness of composites.

Flexural strength and Vickers hardness have obeyed the same trend observed with the fracture toughness, Figs. 10 (a and b). They increased with the addition of Pr_2_O_3_ up to 0.75 wt%, then they showed a rapid decrease upon adding 1 wt% Pr_2_O_3_. It is noticed that the flexural strength was dramatically decreased compared to the hardness decrease. The enhancement of the mechanical properties of the P1 and P2 samples can be attributed to an increase in the samples’ densification behavior compared to the P3 sample. As the ceramic body became denser, the porosity content decreased. Ceramics show very little or no macroscopic plastic deformation before they fracture, meaning they cannot absorb the energy transferred to them. Also, porosity creates stress concentration points. When stress reaches a critical level, a crack forms. Once the crack originates, it spreads until it fractures. Additionally, porosity reduces the cross-sectional area on which a load can be applied, thereby decreasing the stress the ceramic bodies can withstand^31^.

The enhancement in Vickers hardness can be attributed to the addition of nano-sized Pr_2_O_3_ ions, as they enriched the coherence between the grains, which reflected on the sample’s hardness. In addition, the coefficient of thermal expansion dissimilarities between the body components (ZrO_2_: 10.8 × 10^− 6^/°C, Al_2_O_3_: 8.8 × 10^− 6^/°C, Pr_2_O_3_: 10.7 × 10^− 6^/°C and Mullite: 5.4 × 10^− 6^/°C, at 0–1000 °C) presented interior stresses in the ceramic bodies, which enhances the Vickers hardness^32^.

## Conclusions

1. Bulk density of M0 composite increased with increasing sintering temperatures from 1550 to 1650 °C, while apparent porosity decreased.
2. Bulk density of studied composites increased with increasing Pr_2_O_3_ content up to 0.75 weight%, then decreased with further increasing of Pr_2_O_3_ content.
3. Sintering at 1600 °C was chosen as the sintering temperature for all studied samples for comparison reasons.
4. Small amounts of Pr_2_O_3_ up to 0.75 weight% improved the mechanical properties of the studied composites, while the addition of 1 weight% Pr_2_O_3_ rapidly decreases the mechanical properties.
5. The increase in the m-ZrO_2_ content increases the fracture toughness through the transformation toughening mechanism. The other factors enhancing the fracture toughness of the samples are the formation of anisotropic elongated Al_2_O_3_ grains, which can generate toughening mechanisms such as crack bridging and crack deflection. While the presence of Pr_2_O_3_ enhanced the fracture toughness through the creation of dislocation lines, dislocation rings, dislocation twisting and sliding in the Al_2_O_3_ matrix.

## Data Availability

All data supporting the findings of this study are available within the paper.

## References

[1] Heimann, R. B. *Classic and Advanced Ceramics: From Fundamentals to Applications* (Wiley, 2010).

[2] Sarker, S., Mumu, H. T., Al-Amin, M., Alam, M. Z. & Gafur, M. Impacts of inclusion of additives on physical, microstructural, and mechanical properties of Alumina and Zirconia toughened alumina (ZTA) ceramic composite: A review. *Mater. Today Proc*. **62**, 2892–2918. 10.1016/j.matpr.2022.02.481 (2022).

[3] Dhar, S. A., Mumu, H. T., Sarker, S. & Rashid, A. B. Influences of sintering time on the structures and mechanical properties of zirconia toughened alumina nanocomposites. *Mater. Today Proc*. **44**, 1356–1360. 10.1016/j.matpr.2020.11.504 (2021).

[4] Naga, S., Awaad, M., Bondioli, F., Fino, P. & Hassan, A. Thermal diffusivity of ZTA composites with different YSZ quantity. *J. Alloys Compd.* **695**, 1859–1862. 10.1016/j.jallcom.2016.11.019 (2017).

[5] Hofer, A. K. et al. Effect of second phase addition of zirconia on the mechanical response of textured alumina ceramics. *J. Eur. Ceram. Soc.* **43**, 2935–2942. 10.1016/j.jeurceramsoc.2022.08.058 (2023).

[6] Guo, R., Guo, D., Chen, Y., Yang, Z. & Yuan, Q. In situ formation of LaAl11O18 rodlike particles in ZTA ceramics and effect on the mechanical properties. *Ceram. Int.* **28**, 699–704. 10.1016/S0272-8842(02)00031-7 (2002).

[7] Ismaila, H., Zarawi, N. I. & Mohamad, H. Effect of titanium dioxide and niobium pentoxide addition on the properties of zirconia toughened alumina added with MgO (ZTA-MgO) ceramic composite. *J. Phys. Conf. Ser*. **2907**, 012021. 10.1088/1742-6596/2907/1/012021 (2024).

[8] Sktani, Z. D. I., Rejab, N. A., Rosli, A. F. Z., Arab, A. & Ahmad, Z. A. Effects of La2O3 addition on microstructure development and physical properties of harder ZTA-CeO2 composites with sustainable high fracture toughness. *J. Rare Earths*. **39**, 844–849. 10.1016/j.jre.2020.06.005 (2021).

[9] Sktani, Z. D. I., Rejab, N. A. & Ahmad, Z. A. Tougher and harder zirconia toughened alumina (ZTA) composites through in situ microstructural formation of LaMgAl11O19. *Int. J. Refract. Met. Hard Mater.* **79**, 60–68. 10.1016/j.ijrmhm.2018.11.009 (2019).

[10] Maiti, K. & Sil, A. Microstructural relationship with fracture toughness of undoped and rare earths (Y, La) doped Al2O3–ZrO2 ceramic composites. *Ceram. Int.* **37**, 2411–2421. 10.1016/j.ceramint.2011.05.089 (2011).

[11] Xu, X. et al. Preparation and thermal shock resistance investigation of ZTA-La_2_O_3_ composite ceramics for porous medium combustion materials. *Ceram. Int.* **49**, 18645–18653. 10.1016/j.ceramint.2023.02.241 (2023).

[12] Weixin, L., Mingmin, B. & Mingyu, B. Effect of PrAlO_3_ on mechanical properties of zirconia toughened alumina bioceramics after hydrothermal aging. *J. Chin. Ceramic Soc.* **51**, 721–729 (2023). https://cstr.cn/32186.14

[13] Zhang, X., Zhu, D. & Liang, J. Effect of in-situ formed whiskers diameter from tourmaline on microstructure and mechanical properties of 3Y-TZP ceramics. *Ceram. Int.* **49**, 236–242. 10.1016/j.ceramint.2022.08.336 (2023).

[14] Emam, W. et al. Controlling polymorphic structures and investigating electric properties of Ca-doped zirconia using solid state ceramic method. *J. Solid State Chem.* **228**, 153–159. 10.1016/j.jssc.2015.03.009 (2015).

[15] Kröger, F. Electronic conductivity of calcia-stabilized zirconia. *J. Am. Ceram. Soc.* **49**, 215–218. 10.1111/j.1151-2916.1966.tb13237.x (1966).

[16] Xue, J. & Dieckmann, R. Variation of the oxygen content in tetragonal, calcium oxide-doped zirconia. *Solid State Ionics*. **73**, 273–282. 10.1016/0167-2738(94)90044-2 (1994).

[17] Chen, R. & Tuan, W. Toughening alumina with silver and zirconia inclusions. *J. Eur. Ceram. Soc.* **21**, 2887–2893. 10.1016/S0955-2219(01)00230-8 (2001).

[18] Zhang, N. & Zaeem, M. A. Competing mechanisms between dislocation and phase transformation in plastic deformation of single crystalline yttria-stabilized tetragonal zirconia nanopillars. *Acta Mater.* **120**, 337–347. 10.1016/j.actamat.2016.08.075 (2016).

[19] Hoch, M. Lattice defects in tetragonal ZrO2. *J. Am. Ceram. Soc.* **47**, 630–632. 10.1111/j.1151-2916.1964.tb13122.x (1964).

[20] Deligiannakis, Y., Mantzanis, A., Zindrou, A., Smykala, S. & Solakidou, M. Control of monomeric Vo’s versus Vo clusters in ZrO2 – x for solar-light H2 production from H2O at high-yield (millimoles gr ^–1 ^h^–1^). *Sci. Rep.* **12**, 15132–15132. 10.1038/s41598-022-19382-3[ (2022). 36071088 10.1038/s41598-022-19382-3 PMC9452565

[21] Li, P., Chen, I. W. & Penner-Hahn, J. E. Effect of dopants on zirconia stabilization—An X‐ray absorption study: I, trivalent dopants. *J. Am. Ceram. Soc.* **77**, 118–128. 10.1111/j.1151-2916.1994.tb06964.x (1994).

[22] Li, P., Chen, I. W. & Penner-Hahn, J. E. Effect of dopants on zirconia stabilization—An X‐ray absorption study: II, tetravalent dopants. *J. Am. Ceram. Soc.* **77**, 1281–1288. 10.1111/j.1151-2916.1994.tb05403.x (1994).

[23] Hassan, A., Naga, S. & Awaad, M. Toughening and strengthening of Nb2O5 doped zirconia/alumina (ZTA) composites. *Int. J. Refract. Met. Hard Mater.* **48**, 338–345. 10.1016/j.ijrmhm.2014.10.006 (2015).

[24] Kibbel, B. & Heuer, A. H. Exaggerated grain growth in ZrO2-toughened Al2O3. *J. Am. Ceram. Soc.* **69**, 231–236. 10.1111/j.1151-2916.1986.tb07414.x (1986).

[25] Naga, S. M., Ahmed, M. A., Sayed, A. Z., Farag, R. S. & Abdelmegeed, A. F. Effect of ceria on the properties of ceria stabilized zirconia/alumina/ceria (ZTA/Ce) composites. *Elixir Appl. Chem.* **102**, 44354–44358. https://share.google/3HtNGzdz6n2WpjHr2 (2017).

[26] Belles, L. & Deligiannakis, Y. Control of the tetragonal versus monoclinic phases of ZrO2 through flame spray pyrolysis. *Powder Technol.* **464**, 121207. 10.1016/j.powtec.2025.121207 (2025).

[27] Lee, J. H. et al. Grain and grain boundary activities observed in alumina–zirconia–magnesia spinel nanocomposites by in situ nanoindentation using transmission electron microscopy. *Acta Mater.* **58**, 4891–4899. 10.1016/j.actamat.2010.05.027 (2010).

[28] Chen, T., Mohamed, F. A. & Mecartney, M. L. Threshold stress superplastic behavior and dislocation activity in a three-phase alumina–zirconia–mullite composite. *Acta Mater.* **54**, 4415–4426. 10.1016/j.actamat.2006.05.002 (2006).

[29] Tochigi, E., Miao, B., Kondo, S., Shibata, N. & Ikuhara, Y. TEM characterization of lattice defects associated with deformation and fracture in α-Al2O3. In *The Plaston Concept: Plastic Deformation in Structural Materials*. 133–156. 10.1007/978-981-16-7715-1_7 (Springer, 2022).

[30] Naga, S., Hassan, A., Awaad, M. & Bondioli, F. Influence of Ta2O5 doping on the microstructure, physical and mechanical properties of a-alumina ceramics. *J. Ceram. Sci. Technol.* **4**, 187–192. 10.4416/JCST2013-00017 (2013).

[31] Rejab, N. A., Azhar, A. Z. A., Ratnam, M. M. & Ahmad, Z. A. The effects of CeO2 addition on the physical, microstructural and mechanical properties of yttria stabilized zirconia toughened alumina (ZTA). *Int. J. Refract. Met. Hard Mater.* **36**, 162–166. 10.1016/j.ijrmhm.2012.08.010 (2013).

[32] Naga, S., Hassan, A. & Awaad, M. Physical and mechanical properties of Ta2O5 doped zirconia-toughened alumina (ZTA) composites. *Ceram. Int.* **41** 6248–6255. 10.1016/j.ceramint.2015.01.039 (2015).

